# A new species of *Cyphomyia* Wiedemann from the Dominican Republic with a key to Caribbean species of the genus (Diptera, Stratiomyidae, Clitellariinae)

**DOI:** 10.3897/zookeys.453.8623

**Published:** 2014-11-11

**Authors:** Norman E. Woodley

**Affiliations:** 1 Systematic Entomology Laboratory, Beltsville Agricultural Research Center–ARS–USDA, ℅ Smithsonian Institution NHB-168, P. O. Box 37012, Washington, DC 20013-7012, USA

**Keywords:** Neotropical Region, Caribbean, Dominican Republic, taxonomy, new species

## Abstract

A new species of *Cyphomyia* Wiedemann, *Cyphomyia baoruco* **sp. n.**, is described from the Dominican Republic. A key to the species of *Cyphomyia* known from the Caribbean islands is provided.

## Introduction

The genus *Cyphomyia* Wiedemann is a fairly large genus of Stratiomyidae with 83 species. A large majority of the species are found in the Neotropical Region where 72 species are known (Woodley 2001). Eight species have been described from Caribbean islands. Only *Cyphomyia marginata* Loew, described from Cuba, is also known to occur on a mainland area, southern Florida. The Caribbean species have never been systematically revised and have remained difficult to identify without comparative material. Seven of the eight species are quite similar in general appearance, being bluish to bluish black in color with three more or less distinct silvery pilose vittae on the scutum, silvery pilose spots on the abdomen, and nearly hyaline wings. Males have conspicuously, densely pilose eyes, and females of some of the species have shorter pilosity on the eyes or have it very reduced. This is an unusual character state in the genus as most mainland species have bare eyes.

The purpose of this paper is to describe a new species of *Cyphomyia* from the Dominican Republic that differs in general appearance from the described Caribbean species, having a bluish black body with a weakly vittate scutum, no silvery spots on the abdomen, and particularly by having dark wings. This appearance is similar to many mainland species of *Cyphomyia*. In addition, a key is presented that includes all of the described Caribbean species of *Cyphomyia*.

## Methods

Specimens examined in this study are all housed in the Department of Entomology, National Museum of Natural History, Smithsonian Institution, Washington, DC, USA (USNM). Images of the primary types of *Cyphomyia acuminata* James, *Cyphomyia brevis* James, *Cyphomyia marginata* Loew, and *Cyphomyia rubra* Loew were examined on the Museum of Comparative Zoology, Harvard University, type database internet site (Museum of Comparative Zoology 2014).

Specimens were examined using a Zeiss Stemi SV 11 stereomicroscope. Male terminalia were dissected and cleared using hot KOH, neutralized with acetic acid, rinsed in water, and are stored in a microvial with glycerin on the specimen pin.

## Results

### Cyphomyia baoruco

Taxon classificationAnimaliaDipteraStratiomyidae

Woodley sp. n.

http://zoobank.org/E5019831-62C9-4150-AD82-44E98F71B60C

Figs 1 –6

#### Diagnosis.

*Cyphomyia baoruco* can be separated from all other Caribbean species of *Cyphomyia* by its bluish black body, the abdomen without silvery pilose spots dorsally, and its darkly infuscated wings. The male (female unknown) can be separated from New World mainland species by its eyes that have very long pilosity that appears slightly crinkly. Mainland species with pilose eyes generally have hairs that are shorter and denser, and none have crinkly pilosity.

#### Description.

**Figure 1.**
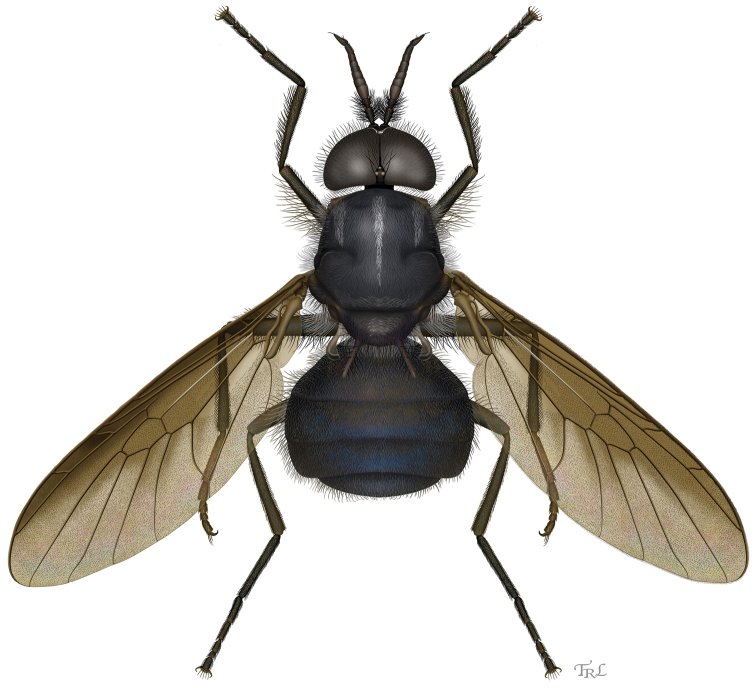
Dorsal habitus of *Cyphomyia baoruco* Woodley, sp. n..

**Figures 2–4.**
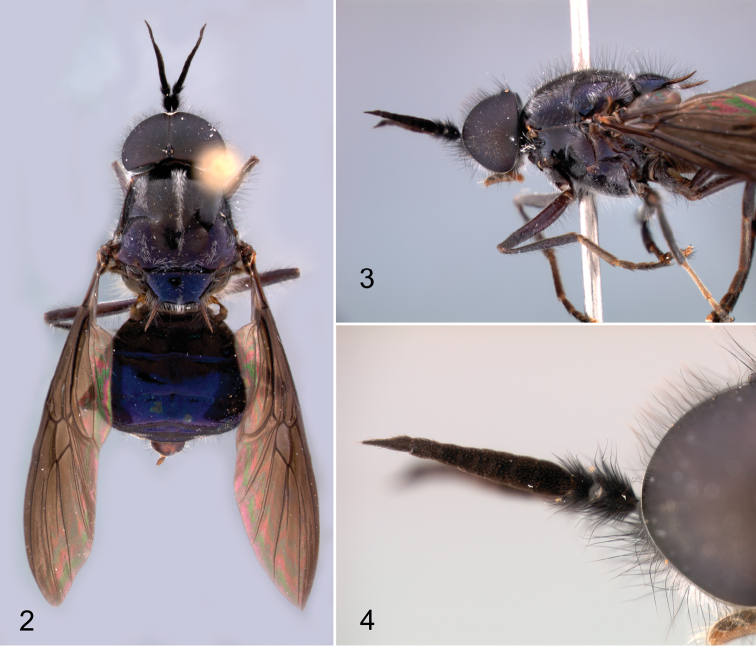
Photographic images of the holotype male of *Cyphomyia baoruco* Woodley, sp. n. **2** Dorsal habitus **3** Head and thorax, left lateral view **4** Anterior part of head and antenna, left lateral view.

**Male.** (Figs 1–2). *Head*: Black, frontal triangle vaguely more brownish; eyes large, holoptic on upper frons, ommatidia nearly uniform in size without abrupt transition, with moderately dense, black pilosity that is about the length of scape and slightly crinkly (Figs 3–4); face very slightly convex, evenly receding to oral margin; postocular orbit, only narrowly visible in lower half in profile; head largely devoid of tomentum, present only along lower part of postocular orbit and extremely narrow strip along eye margin on gena, face, and frons, where it is grayish; head with very long pilosity, about length of scape+pedicel, of moderate density, black on ocellar triangle, face, and gena, with some whitish hairs intermixed on lower part of face and entirely whitish on lower gena and postgena; antenna (Fig. 4) black, 1.71 times length of head, gradually tapering from base to apex, ratio of segments 19:9:91[16:10:7:6:8:10:12:22], last flagellomere acuminate, scape and pedicel very densely set with bushy black pilosity, some hairs almost as long as scape+pedicel, flagellum velvety tomentose, a few short hairs present on apical flagellomere; palpus black, small, two-segmented (mostly obscured by labellum); proboscis dark yellowish, brownish laterally on labellum.

*Thorax*: Black, vaguely browner under wing base, postpronotal lobe, lateral part of postalar callus, and scutellar spines brownish yellow; scutellum with spines a little longer than scutellum, almost in same plane as scutellum, curving very slightly upward; prosternum and medial part of laterotergite with yellowish white tomentum, small areas on meron with sparser, inconspicuous tomentum; pilosity of thorax primarily silvery white, partly semi-appressed on scutum where it forms a narrow medial vitta that decreases in width posteriorly so that it is only a few hairs wide near scutellum, and sublateral patches forming wider, more poorly developed vittae anterior to transverse suture; other areas of scutum with semi-appressed dark hairs, and both scutum and scutellum including spines with very long, erect, slightly wavy black hairs (Fig. 3); central part of anepisternum, most of meron, and medial part of subscutellum bare and shiny; legs dark brown to brownish black, front and middle tarsi with basal two tarsomeres paler, dark yellowish; legs mostly pilose, a mixture of pale and dark hairs, mostly short and semi-appressed, longer erect hairs present on posterior and ventral surfaces of front and middle femora, most of hind femur, and posterior surfaces of front and middle tibiae; wing with dark brown infuscation over entire surface that gradually gets paler posteriorly, entirely set with microtrichia except for strip along anterior portion of cell cup and most of alula; halter with stem yellowish, knob brown.

*Abdomen*: Dark brownish with slight bronzy reflections, subshiny on tergites 1 and 2 and basal part of tergite 3, remainder black with distinct metallic blue reflections, shiny; sternite 1 brown, uniformly set with brownish tomentum, 2–5 black with metallic blue reflections, shiny, segments beyond 5 brownish; tergites with dark, semi-appressed pilosity, with longer erect hairs laterally, some of which are whitish on basal two tergites, becoming progressively shorter posteriorly; first two sternites with erect, whitish hairs medially, otherwise sternites with short, semi-appressed pilosity.

**Figures 5–6.**
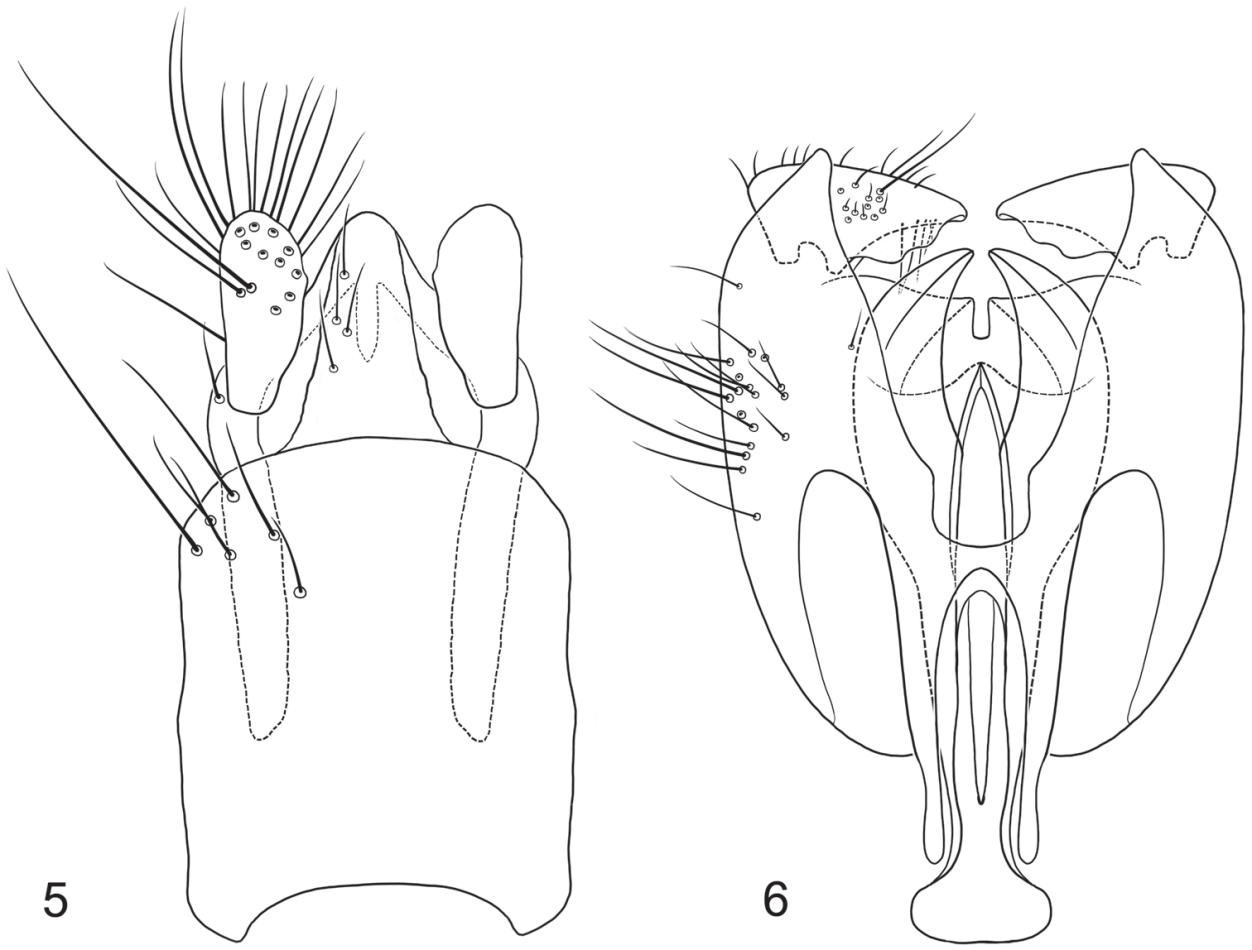
Male terminalia of *Cyphomyia baoruco* Woodley, sp. n. **5** Epandrium and postgenital segments, dorsal view **6** Genital capsule and phallic complex, dorsal view.

*Male terminalia*: With gonocoxites (Fig. 6) slightly longer than wide with lateral triangular processes covering gonostylar articulation, gonocoxal apodemes extending anteriorly beyond anterior margin of genital capsule; hypandrium completely fused, posterior portion of ventral bridge grooved medially, sharply bilobed; gonostylus with weakly developed rounded process posterolaterally, dorsal edge sharp, slightly produced; phallic complex complicatedly fused with gonocoxites, apparently trifid, medial lobe sharply pointed, apparent lateral lobes longer than medial lobe, flattened and with medial curvature posteriorly; epandrium (Fig. 5) simple, more or less quadrate, a little longer than wide, posterior margin evenly rounded; cercus short, slightly widened and rounded posteriorly.

*Measurements*: Length exclusive of antennae, 9.1 mm; antennal length, 2.5 mm; wing length, 9.5 mm.

**Female.** Unknown.

#### Distribution.

Known only from the Dominican Republic on the island of Hispaniola.

#### Type material.

Holotype male (USNM), **DOMINICAN REPUBLIC:** Pedernales Province, Parque Nacional Sierra de Baoruco, Las Abejas, 18°09.011'N, 71°37.342'W, 1150 meters, 17 July 2006, N.E. Woodley. A Smithsonian Institution barcode label is attached to the specimen: USNMENT 01028720. The holotype is in excellent condition.

#### Etymology.

The species epithet, *baoruco*, is a noun in apposition from the name of the mountain range, Sierra de Baoruco, where the holotype specimen was collected.

#### Remarks.

As noted in the introduction, this species differs from all other Caribbean species of *Cyphomyia* in general appearance. It looks more like many Central and South American species that have a dark bluish black body and dark brown wings. The females of these mainland species often have a bright yellow head, and it will be interesting to see if the female of *Cyphomyia baoruco* also has this feature when it is discovered.

The type locality of *Cyphomyia baoruco*, Las Abejas, is at the southwestern end of the Sierra de Baoruco range, which is more or less continuous with the Massif de la Selle in eastern Haiti. The habitat at this site is classified as premontane wet forest (Fisher-Meerow and Judd 1989), an epiphyte-rich diverse hardwood forest which occurs in a thin strip along the southern part of the mountain range mostly between 1100–1200 meters. Las Abejas has been a fairly well-known collecting site since at least the early 1980s, and has produced some remarkable new species (e.g., Woodley 1993, Konstantinov and Chamorro-Lacayo 2006). This habitat in this region is critically endangered (León et al. 2013). Even though this habitat type is now largely within Parque Nacional Sierra de Baoruco, it has suffered extensive deforestation primarily for subsistence farming, and this is probably more intense at its western end near Haiti. I first visited Las Abejas in 1984 and last in 2006 and the extent of deforestation at the site during that time span was significant.

I have composed a key to the described species of *Cyphomyia* found on Caribbean islands that is provided below. It should be noted, however, that due to the paucity of collecting on many islands in the region, it is likely that additional undescribed species will be found. After the key a few brief notes on the included species are given.

### Key to Caribbean species of *Cyphomyia*

**Table:** 

1	Entire body including legs yellowish to brownish; antenna black; only known from Cuba	***Cyphomyia rubra* Loew**
–	Body predominantly blackish with metallic bluish reflections; antenna variable, but usually with some reddish to yellowish coloration especially basally; Cuba and elsewhere	**2**
2(1)	Scutellum entirely yellowish to brownish red, occasionally vaguely darker at extreme base	**3**
–	Scutellum black, usually with metallic bluish reflections, at most narrow apical margin and spines yellowish	**4**
3(2)	Posterior margin of fifth abdominal tergite with broad yellowish margin, in females this tergite can be mostly yellowish; femora usually yellowish, if brownish the darker coloration is not sharply delimited and is basal, grading into broadly yellowish apex; USA: Florida, Bahamas, Cuba, Puerto Rico	***Cyphomyia marginata* Loew**
–	Posterior margin of fifth abdominal tergite dark; femora and tibiae dark brown to blackish, with joint between them narrowly pale yellow that is sharply delimited; Hispaniola, ?Jamaica	***Cyphomyia albomaculata* (Macquart)**
4(2)	Wing darkly infuscated over entire surface; abdominal tergites without silvery pilosity; Dominican Republic	***Cyphomyia baoruco* sp. n.**
–	Wing hyaline; abdominal tergites with silvery pilosity moderately developed into spots, at least on fifth tergite	**5**
5(4)	Apex of scutellum yellow, visible in dorsal view; Jamaica	***Cyphomyia acuminata* James**
–	Apex of scutellum dark, concolorous with disc, margin may be yellowish brown to brown but this color not visible in dorsal view	**6**
6(5)	All femora yellowish to brownish yellow; Cuba	***Cyphomyia brevis* James**
–	All femora dark brown to black	**7**
7(6)	Scutum above notopleural suture with dark hairs, contrasting with the pale hairs that form the sublateral vittae, so that the anterior end of each vitta is more or less distinct and does not appear to coalesce with lateral pilosity; St. Vincent, Grenada	***Cyphomyia lasiophthalma* Williston**
–	Scutum above notopleural suture with pale hairs, concolorous with those that form the sublateral vitta, so that the anterior end of each vitta is not distinct as it coalesces with lateral pilosity	**8**
8(7)	Basal three flagellomeres uniformly orange, sharply contrasting with darker, more distal flagellomeres, rarely the basal three with a small amount of brownish infuscation; lateral silvery hair patches on tergites 4 and 5 moderately well developed, more or less evident to the naked eye; Puerto Rico, Virgin Islands, St. Kitts, Antigua, St. Lucia	***Cyphomyia chalybea* (Wiedemann)**
–	Basal three flagellomeres more brownish, not sharply contrasting with more distal flagellomeres, if some orangish color present, color evenly grades toward darker apex; lateral silvery hair patches on tergites 4 and 5 poorly developed, not readily evident to naked eye; Dominica	***Cyphomyia dominicana* James**

### Identification notes on Caribbean *Cyphomyia* species

In this section a few brief notes are given concerning identifications and distributions of Caribbean *Cyphomyia*. Full nomenclatural details and synonymy are presented in Woodley (2001) so are not repeated here.

***Cyphomyia acuminata* James.** This species is only known from Jamaica, and I am not aware of any specimen records beyond the original type series. Although some Caribbean *Cyphomyia* have a scutellar margin that is lighter in color than the disc, this is the only species that has yellowish color between the spines that extends onto the disc and can be seen in dorsal view.

***Cyphomyia albomaculata* (Macquart).** This species was described from Haiti. It is widespread and common at lower elevations in the Dominican Republic. Lindner (1949) recorded the species from Jamaica. The few specimens I have seen from Jamaica seem to be this species but have a paler basitarsus on the hind leg that is not apically blackish as found in Hispaniolan specimens.

***Cyphomyia brevis* James.** This species is known only from Cuba. Because it has pale femora, this species is incorrectly placed by James (1940: 128) in his key. In order to get to couplet 22 where it keys out, you have to chose “anterior femora black, brown, or blue; yellow at apex or not at all” at couplet 11, which is incorrect.

***Cyphomyia chalybea* (Wiedemann).** This species is quite similar to *Cyphomyia dominicana*, but is almost always readily distinguishable by the orange color of the basal three antennal flagellomeres. The antennal flagellum is shorter than in *Cyphomyia dominicana*, but this is difficult to appreciate without having both species at hand. Also, *Cyphomyia chalybea* has more conspicuous pilose spots on the abdomen. James (1967: 4) noted these differences when describing *Cyphomyia dominicana*. Additionally, the pilose vittae on the scutum are more conspicuous in *Cyphomyia chalybea*. In USNM there are specimens of this species from the Virgin Islands (St. Thomas, St. Croix, Guana), St. Kitts, Antigua, and St. Lucia. Records from Cuba, Dominican Republic, and Jamaica require confirmation before they can be considered part of the distribution of this species.

***Cyphomyia dominicana* James.** This species is quite similar to *Cyphomyia chalybea*; comparisons are noted above under that species. Part of the type series of this species, as well as a few additional specimens, were reared but unfortunately the larval host was not recorded.

***Cyphomyia lasiophthalma* Williston.** I am basing my concept of this species on the specimens that James (1967: 4) examined from Grenada, which are present in the USNM collection. More material is necessary from the Lesser Antilles to get a more precise idea of species concepts in this region.

***Cyphomyia marginata* Loew.** This species was described from Cuba. I have examined specimens from USA: Florida, the Bahamas, and Puerto Rico that I consider conspecific, although specimens from Puerto Rico generally have darker legs. This species has also been recorded from Jamaica and Hispaniola, but I think that these records almost certainly refer to *Cyphomyia albomaculata*. James (1940) synonymized *Cyphomyia scutellata* (Cresson), described from Costa Rica, with *Cyphomyia marginata*. James noted that the abdomen of *Cyphomyia scutellata* was entirely black so that synonymy must be incorrect.

***Cyphomyia rubra* Loew.** This unusual species is only known from Cuba. Only three other described species of *Cyphomyia*, all from Central or South America, out of 72 Neotropical species, are extensively pale in coloration. The male of this species has not been described and remains unknown.

## Supplementary Material

XML Treatment for Cyphomyia baoruco
